# Supplementary material for: Sex differences in multimorbidity and polypharmacy trends: A repeated cross-sectional study of older adults in Ontario, Canada
Source: PLoS One. 2021 Apr 26;16(4):e0250567. doi: 10.1371/journal.pone.0250567 (PMC8075196; doi:10.1371/journal.pone.0250567)
Supplement: S1 Table — (DOCX) [file pone.0250567.s001.docx]

**S1 Table: Description of Ontario health administrative databases**

| **DATABASE** | **DESCRIPTION** |
| --- | --- |
| Registered Persons Database (RPDB) | Provides basic demographic information (age, sex, area of residence, date of birth, date of death) about anyone who has ever received an Ontario health card number (i.e., enrolled in the provincial publicly funded health insurance system). |
| Postal Code Conversion File (PCCF) | Links neighbourhood-level measures of rurality and income quintile to individuals based on geographic identifiers. |
| CONTACT | Contains data on eligibility for services covered under OHIP. |
| Drug Identification Number (DIN) | This file contains a near exhaustive list of Drug Identification Numbers used in Canada from 1990 forward. |
| Ontario Drug Benefit (ODB) program | Contains claims for prescription drugs received under the Ontario Drug Benefit program, mainly for those age ≥65, nursing home residents and persons receiving social assistance. |
| Canadian Institute for Health Information - Discharge Abstract Database (DAD) | Contains patient-level data (demographic, diagnoses, procedures) for all admissions to acute care hospitals in Ontario. |
| Canadian Institute for Health Information – National Ambulatory Care Reporting System (NACRS) | Contains patient-level data (demographic, diagnoses, procedures) for all visits made to hospital and community based ambulatory care centres (emergency departments, day surgery, dialysis, cancer care clinics) in Ontario. |
| Canadian Institute for Health Information - Ontario Mental Health Reporting System (OMHRS) | Contains patient-level data (demographic, mental & physical health, diagnoses, supports and services) for admissions to inpatient mental health care in Ontario. |
| Ontario Health Insurance Plan (OHIP) Claims Database | Contains most claims for physician-services (and others) paid for by the Ontario Health Insurance Plan. |
