# Supplementary material for: Sex differences in multimorbidity and polypharmacy trends: A repeated cross-sectional study of older adults in Ontario, Canada
Source: PLoS One. 2021 Apr 26;16(4):e0250567. doi: 10.1371/journal.pone.0250567 (PMC8075196; doi:10.1371/journal.pone.0250567)
Supplement: S2 Table — (DOCX) [file pone.0250567.s002.docx]

**S2 Table: Methods for ascertaining chronic conditions and level of multimorbidity**

We identified the presence of 17 chronic conditions for persons >65 years of age in Ontario, Canada on April 1, 2003 and on April 1, 2016 from health administrative databases, including: Discharge Abstract Database (DAD), Ontario Health Insurance Plan (OHIP), Ontario Drug Benefits (ODB), Drug lists (DIN), National Ambulatory Care Reporting System (NACRS), Ontario Mental Health Reporting System (OMHRS) and Registered Persons Database (RPDB).

Conditions included: acute myocardial infarction (AMI), asthma, (any) cancer, cardiac arrhythmia, chronic obstructive pulmonary disease (COPD), congestive heart failure (CHF), chronic coronary syndrome, dementia, diabetes, hypertension, non-psychotic mood and anxiety disorders, other mental illnesses (including schizophrenia, delusions, and other psychoses; personality disorders; and substance abuse), osteoarthritis, osteoporosis, renal failure, rheumatoid arthritis, and stroke (excluding transient ischemic attack). These conditions were selected based on their system burden, in terms of population prevalence [1] and economic costs [2] and have been used in multiple research studies of multimorbidity in Ontario [1,3-7]. Similar sets of conditions have been used in multimorbidity research elsewhere [8-9]. Where applicable we used validated algorithms to ascertain cases (AMI, asthma, CHF, COPD, dementia, diabetes, hypertension and rheumatoid arthritis) [10-17]. All other conditions were defined based on the presence of any one inpatient hospital diagnostic code (DAD data) or two or more outpatient physician billing codes (OHIP data) within a 2-year period using relevant ICD-9 and ICD-10 codes. The earliest utilization date satisfying the algorithm requirement was used to identify incident cases.

From this data we defined chronic disease burden based on a simple count of prevalent conditions, which was coded as zero/one, two, three, four, or five-plus conditions. This method was used because there are currently no methods for creating meaningful clusters of chronic conditions, particularly where no central index condition is present.

| **Condition [reference for validated algorithm]** | **ICD 9 / OHIP** | **ICD 10** | **Drug Subclass (ODB)*** |
| --- | --- | --- | --- |
| Acute Myocardial Infarction (AMI) [10] | 410 | I21 |  |
| Osteo- and other Arthritis: |  |  |  |
| (A) Osteoarthritis | 715 | M15-M19 |  |
| (B) Other Arthritis (includes Synovitis, Fibrositis, Connective tissue disorders, Ankylosing spondylitis, Gout Traumatic arthritis, pyogenic arthritis, Joint derangement, Dupuytren’s contracture, Other MSK disorders) | 727, 729, 710, 720, 274, 716, 711, 718, 728, 739 | M00-M03, M07, M10, M11-M14, M20-M25, M30-M36, M65-M79 |  |
| Arthritis - Rheumatoid arthritis [17] | 714 | M05-M06 |  |
| Asthma [11] | 493 | J45, J46 |  |
| (all) Cancers | 140-239 | C00-C26, C30-C44, C45-C97 |  |
| Cardiac Arrhythmia | 427 (OHIP) / 427.3 (DAD) | I48.0, I48.1 |  |
| Congestive Heart Failure [15] | 428 | I500, I501, I509 |  |
| Chronic Obstructive Pulmonary Disease [12] | 491, 492, 496 | J41, J42, J43, J44 |  |
| Coronary syndrome (excluding AMI) | 411-414 | I20, I22-I25 |  |
| Dementia [14] | 290, 331 (OHIP) / 046.1, 290.0, 290.1, 290.2, 290.3, 290.4, 294, 331.0, 331.1, 331.5, F331.82^†^ (DAD) | F00, F01, F02, F03, G30 | Cholinesterase Inhibitors |
| Diabetes [13] | 250 | E10, E11, E13, E14 | Oral anti-glycemics, Insulin, Anti-diabetic agents: Misc |
| Hypertension [16] | 401, 402, 403, 404, 405 | I10, I11, I12, I13, I15 |  |
| (Other) Mental Illnesses | 291, 292, 295, 297, 298, 299, 301, 302, 303, 304, 305, 306, 307, 313, 314, 315, 319 | F04, F050, F058, F059, F060, F061, F062, F063, F064, F07, F08, F10, F11, F12, F13, F14, F15, F16, F17, F18, F19, F20, F21, F22, F23, F24, F25, F26, F27, F28, F29, F340, F35, F36, F37, F430, F439, F453, F454, F458, F46, F47, F49, F50, F51, F52, F531, F538, F539, F54, F55, F56, F57, F58, F59, F60, F61, F62, F63, F64, F65, F66, F67, F681, F688, F69, F70, F71, F72, F73, F74, F75, F76, F77, F78, F79, F80, F81, F82, F83, F84, F85, F86, F87, F88, F89, F90, F91, F92, F931, F932, F933, F938, F939, F94, F95, F96, F97, F98 |  |
| Mood, anxiety, depression and other nonpsychotic disorders | 296, 300, 309, 311 | F30, F31, F32, F33, F34 (excl. F34.0), F38, F39, F40, F41, F42, F43.1, F43.2, F43.8, F44, F45.0, F45.1, F45.2, F48, F53.0, F68.0, F93.0, F99 |  |
| Osteoporosis | 733 | M81, M82 |  |
| Renal failure | 403, 404, 584, 585, 586, v451 | N17, N18, N19, T82.4, Z49.2, Z99.2 |  |
| Stroke (excluding transient ischemic attack) | 430, 431, 432, 434, 436 | I60-I64 |  |
| NOTES: | | | |
| Abbreviations: ICD = International Classification of Disease; ODB = Ontario Drug Benefit program database; OHIP = Ontario Health Insurance Plan, physician billings database; | | | |
| All case definitions use all available retrospective health administrative data ascertain disease status, with the exception of AMI (1 year prior to index), Cancer (2 years), Mood Disorder (2 years) and Other Mental Illnesses (2 years) | | | |
| AMI, Asthma, COPD, CHF, Dementia, Diabetes, Hypertension, and Rheumatoid Arthritis are based on validated case algorithms (see Sources 10-17 below, respectively). All other conditions required at least one diagnosis recorded in acute care (CIHI) or two diagnoses recorded in physician billings within a two-year period. | | | |
| *ODB prescription drug records are not available for the majority of persons under the age of 65 | | | |
| ^†^ For ICD-9 diagnoses captured to the 5th digit, the ICD-9 code of 331.82 (Dementia with Lewy bodies) should be included to define hospital separations. This code is not captured within ICES databases, so its inclusion will not identify additional cases in Ontario | | | |

**References:**

1. Koné Pefoyo AJ, Bronskill SE, Gruneir A, Calzavara A, Thavorn K, Petrosyan Y, Maxwell CJ, Bai Y, Wodchis WP. The increasing burden and complexity of multimorbidity. BMC Public Health. 2015 Apr 23;15:415.
2. Public Health Agency of Canada. Economic burden of illness in Canada, 2005–2008. Ottawa: Public Health Agency of Canada; 2014 [cited Feb 2017]. http://www.phac-aspc.gc.ca/publicat/ebic-femc/2005-2008/assets/pdf/ebic-femc-2005-2008-eng.pdf.
3. Gruneir A, Bronskill SE, Maxwell CJ, Bai YQ, Kone AJ, Thavorn K, Petrosyan Y, Calzavara A, Wodchis WP. The association between multimorbidity and hospitalization is modified by individual demographics and physician continuity of care: a retrospective cohort study. BMC Health Serv Res. 2016 Apr 27;16:154.
4. Lane NE, Maxwell CJ, Gruneir A, Bronskill SE, Wodchis WP. Absence of a socioeconomic gradient in older adults’ survival with multiple chronic conditions. EBioMedicine. 2015 Nov 18;2(12):2094-100.
5. Mondor L, Maxwell CJ, Bronskill SE, Gruneir A, Wodchis WP. The relative impact of chronic conditions and multimorbidity on health-related quality of life in Ontario long-stay home care clients. Qual Life Res. 2016 Oct;25(10):2619-32.
6. Mondor L, Maxwell CJ, Hogan DB, Bronskill SE, Gruneir A, Lane NE, Wodchis WP. Multimorbidity and healthcare utilization among home care clients with dementia in Ontario, Canada: A retrospective analysis of a population-based cohort. Plos Medicine. 2017 Mar; 14(3): e1002249.
7. Petrosyan Y, Bai YQ, Koné Pefoyo AJ, Gruneir A, Thavorn K, Maxwell CJ, Bronskill SE, Wodchis WP. The Relationship between Diabetes Care Quality and Diabetes-Related Hospitalizations and the Modifying Role of Comorbidity. Can J Diabetes. 2017 Feb;41(1):17-25.
8. Goodman RA, Posner SF, Huang ES, Parekh AK, Koh HK. Defining and measuring chronic conditions: imperatives for research, policy, program, and practice. Prev Chronic Dis. 2013;10:E66. pmid:23618546
9. Lochner K, Goodman R, Posner S, Parekh A. Multiple chronic conditions among Medicare beneficiaries: state-level variations in prevalence, utilization, and cost, 2011. Medicare Medicaid Res Rev. 2013;3(3).
10. Austin PC, Daly PA, Tu JV. A multicenter study of the coding accuracy of hospital discharge administrative data for patients admitted to cardiac care units in Ontario. American Heart Journal 2002;144:290–6.
11. Gershon AS, Wang C, Guan J, Vasilevska-Ristovska J, Cicutto L, To T. Identifying patients with physician-diagnosed asthma in health administrative databases. Can Respir J 2009;16:183–8.
12. Gershon AS, Wang C, Guan J, Vasilevska-Ristovska J, Cicutto L, To T. Identifying Individuals with Physician Diagnosed COPD in Health Administrative Databases. Copd 2009;6:388–94.
13. Hux JE, Ivis F, Flintoft V, Bica A. Diabetes in Ontario: Determination of prevalence and incidence using a validated administrative data algorithm. Diabetes Care 2002;25:512–6.
14. Jaakkimainen RL, Bronskill SE, Tierney MC, Herrmann N, Green D, Young J, et al. Identification of Physician-Diagnosed Alzheimer’s Disease and Related Dementias in Population-Based Administrative Data: A Validation Study Using Family Physicians’ Electronic Medical Records. J Alzheimers Dis. 2016 Aug 10;54(1):337–49
15. Schultz SE, Rothwell DM, Chen Z, Tu K. Identifying cases of congestive heart failure from administrative data: a validation study using primary care patient records. Chronic Diseases and Injuries in Canada 2013;33:160–6.
16. Tu K, Campbell NR, Chen Z-L, Cauch-Dudek KJ, McAlister FA. Accuracy of administrative databases in identifying patients with hypertension. Open Med 2007;1:e18–26.
17. Widdifield J, Bernatsky S, Paterson JM, Tu K, Ng R, Thorne JC, Pope JE, Bombardier C. Accuracy of Canadian health administrative databases in identifying patients with rheumatoid arthritis: a validation study using the medical records of rheumatologists. Arthritis Care Res 2013; 65(10): 1582-1591.
