# Supplementary material for: Sex differences in multimorbidity and polypharmacy trends: A repeated cross-sectional study of older adults in Ontario, Canada
Source: PLoS One. 2021 Apr 26;16(4):e0250567. doi: 10.1371/journal.pone.0250567 (PMC8075196; doi:10.1371/journal.pone.0250567)
Supplement: S3 Table — (DOCX) [file pone.0250567.s003.docx]

**S3 Table: Characteristics of older adults (aged ≥66 years) in Ontario, Canada: 2003 vs 2016**

|  | **Total Population** | |
| --- | --- | --- |
|  | **2003** | **2016** |
|  | N=1,452,180 | N=2,103,995 |
| ***Sociodemographic Characteristics*** |  |  |
| Age (Median, IQR)  66-74  75-84  85+ | 74 (70-80)  747,721 (51.5%)  511,787 (35.2%)  192,672 (13.3%) | 74 (69-80)  1,118,146 (53.1%)  633,261 (30.1%)  352,588 (16.8%) |
| Women  Men | 829,533 (57.1%)  622,647 (42.9%) | 1,155,684 (54.9%)  948,311 (45.1%) |
| Income Quintile 1 (lowest) | 290,353 (20.0%) | 372,012 (17.7%) |
| Income Quintile 2 | 314,187 (21.6%) | 414,108 (19.7%) |
| Income Quintile 3 | 292,541 (20.1%) | 414,427 (19.7%) |
| Income Quintile 4 | 270,213 (18.6%) | 444,760 (21.1%) |
| Income Quintile 5 (highest) | 281,047 (19.4%) | 450,897 (21.4%) |
| Urban resident | 1,235,159 (85.1%) | 1,814,861 (86.3%) |
| Rural resident | 216,130 (14.9%) | 289,087 (13.7%) |
| Long term care resident flag (OHIP/ODB) | 62,630 (4.3%) | 76,275 (3.6%) |
| ***Prevalent Chronic Conditions*** |  |  |
| 0/1 Conditions | 370,596 (25.5%) | 402,967 (19.2%) |
| 2 Conditions | 328,848 (22.6%) | 436,371 (20.7%) |
| 3 Conditions | 296,069 (20.4%) | 450,418 (21.4%) |
| 4 Conditions | 206,461 (14.2%) | 344,908 (16.4%) |
| 5+ Conditions | 250,206 (17.2%) | 469,331 (22.3%) |
| **Multimorbidity (2+ Conditions)** | **1,081,584 (74.5%)** | **1,701,028 (80.8%)** |
| # conditions (Median, IQR) | 3 (1-4) | 3 (2-4) |
| Acute myocardial infarction (AMI) | 9,816 (0.7%) | 8,690 (0.4%) |
| Cardiac arrythmia | 183,832 (12.7%) | 306,308 (14.6%) |
| Asthma | 158,409 (10.9%) | 274,876 (13.1%) |
| Cancer | 268,749 (18.5%) | 463,769 (22.0%) |
| Congestive heart failure (CHF) | 156,835 (10.8%) | 198,329 (9.4%) |
| Chronic obstructive pulmonary disease (COPD) | 151,769 (10.5%) | 190,559 (9.1%) |
| Chronic coronary syndrome | 433,830 (29.9%) | 551,463 (26.2%) |
| Dementia | 92,133 (6.3%) | 166,886 (7.9%) |
| Diabetes | 286,696 (19.7%) | 653,008 (31.0%) |
| Hypertension | 916,615 (63.1%) | 1,489,296 (70.8% |
| Non-psychotic Mood/Anxiety Disorders | 223,254 (15.4%) | 230,698 (11.0%) |
| (Other) Mental Health Conditions | 56,152 (3.9%) | 91,900 (4.4%) |
| Osteoarthritis | 832,001 (57.3%) | 1,407,065 (66.9%) |
| Osteoporosis | 142,505 (9.8%) | 293,044 (13.9%) |
| Renal Disease | 45,670 (3.1%) | 172,771 (8.2%) |
| Rheumatoid Arthritis | 27,424 (1.9%) | 53,547 (2.5%) |
| Stroke | 99,838 (6.9%) | 132,608 (6.3%) |
| ***Drug Outcomes*** |  |  |
| # unique drug names dispensed (Median, IQR)  0  1  2  3  4  5+ | 6 (3-10)  143,609 (9.9%)  85,456 (5.9%)  104,860 (7.2%)  116,685 (8.0%)  122,345 (8.4%)  879,225 (60.5%) | 6 (3-10)  163,470 (7.8%)  116,787 (5.6%)  142,578 (6.8%)  163,205 (7.8%)  174,562 (8.3%)  1,343,393 (63.8%) |
|  |  |  |
| **Polypharmacy (5+ drug names)** | **879,225 (60.5%)** | **1,343,393 (63.8%)** |
| **Hyper-polypharmacy (10+ drug names)** | **365,275 (25.2%)** | **582,746 (27.7%)** |
|  |  |  |
| # drug subclasses dispensed (Median, IQR) | **5 (3-9)** | **6 (3-9)** |
| **Polypharmacy (5+ drug subclasses)** | **845,414 (58.2%)** | **1,306,097 (62.1%)** |
| **Hyper-polypharmacy (10+ drug subclasses)** | **298,025 (20.5%)** | **496,802 (23.6%)** |

**Notes:** N (column %) shown unless otherwise stated. Abbreviations: IQR=interquartile range; OHIP=Ontario Health Insurance Program; ODB=Ontario Drug Benefit database
