# Supplementary material for: Sex differences in multimorbidity and polypharmacy trends: A repeated cross-sectional study of older adults in Ontario, Canada
Source: PLoS One. 2021 Apr 26;16(4):e0250567. doi: 10.1371/journal.pone.0250567 (PMC8075196; doi:10.1371/journal.pone.0250567)
Supplement: S4 Table — (DOCX) [file pone.0250567.s004.docx]

**S4 Table: Results (b, SE) from logistic regression analyses used to make predictions for polypharmacy outcome**

|  | **Women** | |  | **Men** | |
| --- | --- | --- | --- | --- | --- |
| **Variable** | **Unadj Model** | **Adj Model** |  | **Unadj Model** | **Adj Model** |
| year=2003 | REF | REF |  | REF | REF |
| year=2016 | -1.048* (0.000) | -1.031* (0.000) |  | 0.198 (0.051) | 0.198 (0.051) |
| Age (cont) | 0.027* (0.000) | 0.026* (0.000) |  | 0.040* (0.000) | 0.040* (0.000) |
| 0/1 Conditions | REF | REF |  | REF | REF |
| 2 Conditions | 1.894* (0.000) | 1.886* (0.000) |  | 2.314* (0.000) | 2.316* (0.000) |
| 3 Conditions | 3.115* (0.000) | 3.097* (0.000) |  | 3.770* (0.000) | 3.757* (0.000) |
| 4 Conditions | 4.443* (0.000) | 4.409* (0.000) |  | 5.177* (0.000) | 5.156* (0.000) |
| 5+ Conditions | 6.700* (0.000) | 6.632* (0.000) |  | 7.103* (0.000) | 7.057* (0.000) |
| year=2003 # Age | REF | REF |  | REF | REF |
| year=2016 # Age | 0.012* (0.000) | 0.012* (0.000) |  | -0.001 (0.421) | -0.001 (0.467) |
| year=2003 # 0/1 | REF | REF |  | REF | REF |
| year=2003 # 2 | REF | REF |  | REF | REF |
| year=2003 # 3 | REF | REF |  | REF | REF |
| year=2003 # 4 | REF | REF |  | REF | REF |
| year=2003 # 5+ | REF | REF |  | REF | REF |
| year=2016 # 0/1 | REF | REF |  | REF | REF |
| year=2016 # 2 | -0.269* (0.011) | -0.267* (0.011) |  | -0.086 (0.517) | -0.086 (0.517) |
| year=2016 # 3 | -0.310* (0.005) | -0.312* (0.005) |  | -0.306* (0.025) | -0.299* (0.029) |
| year=2016 # 4 | -0.486* (0.000) | -0.494* (0.000) |  | -0.419* (0.009) | -0.416* (0.010) |
| year=2016 # 5+ | -1.447* (0.000) | -1.455* (0.000) |  | -0.768* (0.000) | -0.760* (0.000) |
| 0/1 # Age | REF | REF |  | REF | REF |
| 2 # Age | -0.009* (0.000) | -0.009* (0.000) |  | -0.014* (0.000) | -0.014* (0.000) |
| 3 # Age | -0.015* (0.000) | -0.015* (0.000) |  | -0.023* (0.000) | -0.023* (0.000) |
| 4 # Age | -0.024* (0.000) | -0.024* (0.000) |  | -0.032* (0.000) | -0.032* (0.000) |
| 5+ # Age | -0.040* (0.000) | -0.040* (0.000) |  | -0.044* (0.000) | -0.044* (0.000) |
| year=2003 # 0/1 # Age | REF | REF |  | REF | REF |
| year=2003 # 2 # Age | REF | REF |  | REF | REF |
| year=2003 # 3 # Age | REF | REF |  | REF | REF |
| year=2003 # 4 # Age | REF | REF |  | REF | REF |
| year=2003 # 5+ # Age | REF | REF |  | REF | REF |
| year=2016 # 0/1 # Age | REF | REF |  | REF | REF |
| year=2016 # 2 # Age | 0.003 (0.065) | 0.003 (0.070) |  | 0.001 (0.694) | 0.001 (0.692) |
| year=2016 # 3 # Age | 0.003* (0.027) | 0.003* (0.027) |  | 0.004* (0.045) | 0.004 (0.051) |
| year=2016 # 4 # Age | 0.005* (0.002) | 0.005* (0.002) |  | 0.006* (0.010) | 0.005* (0.011) |
| year=2016 # 5+ # Age | 0.018* (0.000) | 0.018* (0.000) |  | 0.010* (0.000) | 0.010* (0.000) |
| Urban Resident |  | REF |  |  | REF |
| Rural Resident |  | 0.020* (0.000) |  |  | -0.038* (0.000) |
| Income Quintile=1 |  | 0.323* (0.000) |  |  | 0.255* (0.000) |
| Income Quintile=2 |  | 0.235* (0.000) |  |  | 0.186* (0.000) |
| Income Quintile=3 |  | 0.199* (0.000) |  |  | 0.162* (0.000) |
| Income Quintile=4 |  | 0.129* (0.000) |  |  | 0.103* (0.000) |
| Income Quintile=5 |  | REF |  |  | REF |
| Constant | -2.937* (0.000) | -3.075* (0.000) |  | -4.370* (0.000) | -4.473* (0.000) |
| Observations | 1978980 | 1978980 |  | 1565565 | 1565565 |
| **Notes:** |  |  |  |  |  |
| * denotes p<0.05 |  |  |  |  |  |
