# Supplementary material for: Sex differences in multimorbidity and polypharmacy trends: A repeated cross-sectional study of older adults in Ontario, Canada
Source: PLoS One. 2021 Apr 26;16(4):e0250567. doi: 10.1371/journal.pone.0250567 (PMC8075196; doi:10.1371/journal.pone.0250567)
Supplement: S5 Table — (DOCX) [file pone.0250567.s005.docx]

**S5 Table: Marginal probabilities of polypharmacy (5+ drugs) and unadjusted and adjusted risk differences and risk ratios, by select ages, level of multimorbidity and sex (excluding older adults residing in long-term care facilities)**

| **Women** | |  |  |  |  |  |  |
| --- | --- | --- | --- | --- | --- | --- | --- |
| **Age** | **Level of MMB** | **P(2003)** | **P(2016)** | **RD, Unadj** | **RD, Adj** | **RR, Unadj** | **RR, Adj** |
| 70 | 0/1 | 25.8 | 22.1 | -3.76 (-4.06, -3.46)* | -3.53 (-3.82, -3.23)* | 0.85 (0.84, 0.86)* | 0.86 (0.85, 0.87)* |
| 70 | 2 | 55.1 | 47.8 | -7.28 (-7.64, -6.91)* | -6.95 (-7.31, -6.59)* | 0.87 (0.86, 0.87)* | 0.87 (0.87, 0.88)* |
| 70 | 3 | 72.7 | 66.7 | -6.03 (-6.39, -5.66)* | -5.78 (-6.15, -5.42)* | 0.92 (0.91, 0.92)* | 0.92 (0.92, 0.93)* |
| 70 | 4 | 84.7 | 80.1 | -4.57 (-4.95, -4.18)* | -4.42 (-4.81, -4.04)* | 0.95 (0.94, 0.95)* | 0.95 (0.94, 0.95)* |
| 70 | 5+ | 94.2 | 91.8 | -2.38 (-2.64, -2.12)* | -2.35 (-2.61, -2.09)* | 0.97 (0.97, 0.98)* | 0.98 (0.97, 0.98)* |
| 80 | 0/1 | 30.6 | 28.7 | -1.88 (-2.30, -1.46)* | -1.61 (-2.03, -1.19)* | 0.94 (0.93, 0.95)* | 0.95 (0.93, 0.96)* |
| 80 | 2 | 59.1 | 55.1 | -3.98 (-4.37, -3.58)* | -3.69 (-4.08, -3.30)* | 0.93 (0.93, 0.94)* | 0.94 (0.93, 0.94)* |
| 80 | 3 | 74.7 | 71.5 | -3.15 (-3.48, -2.82)* | -2.94 (-3.27, -2.61)* | 0.96 (0.95, 0.96)* | 0.96 (0.96, 0.96)* |
| 80 | 4 | 85.0 | 82.4 | -2.59 (-2.89, -2.29)* | -2.45 (-2.76, -2.15)* | 0.97 (0.97, 0.97)* | 0.97 (0.97, 0.97)* |
| 80 | 5+ | 93.4 | 92.6 | -0.81 (-0.99, -0.63)* | -0.77 (-0.95, -0.59)* | 0.99 (0.99, 0.99)* | 0.99 (0.99, 0.99)* |
| 90 | 0/1 | 35.9 | 36.5 | 0.62 (-0.28, 1.52) | 0.90 (0.00, 1.79)* | 1.02 (0.99, 1.04) | 1.03 (1.00, 1.05)* |
| 90 | 2 | 62.9 | 62.1 | -0.77 (-1.51, -0.04)* | -0.53 (-1.27, 0.20) | 0.99 (0.98, 1.00)* | 0.99 (0.98, 1.00) |
| 90 | 3 | 76.6 | 76.0 | -0.62 (-1.22, -0.03)* | -0.45 (-1.05, 0.15) | 0.99 (0.98, 1.00)* | 0.99 (0.99, 1.00) |
| 90 | 4 | 85.3 | 84.5 | -0.82 (-1.38, -0.26)* | -0.69 (-1.25, -0.12)* | 0.99 (0.98, 1.00)* | 0.99 (0.99, 1.00)* |
| 90 | 5+ | 92.6 | 93.3 | 0.78 (0.43, 1.12)* | 0.83 (0.49, 1.18)* | 1.01 (1.00, 1.01)* | 1.01 (1.01, 1.01)* |
| **Men** | |  |  |  |  |  |  |
| **Age** | **Level of MMB** | **P(2003)** | **P(2016)** | **RD, Unadj** | **RD, Adj** | **RR, Unadj** | **RR, Adj** |
| 70 | 0/1 | 17.5 | 19.3 | 1.79 (1.52, 2.07)* | 1.91 (1.63, 2.18)* | 1.10 (1.09, 1.12)* | 1.11 (1.09, 1.13)* |
| 70 | 2 | 44.9 | 47.0 | 2.07 (1.67, 2.47)* | 2.26 (1.86, 2.66)* | 1.05 (1.04, 1.06)* | 1.05 (1.04, 1.06)* |
| 70 | 3 | 65.1 | 66.8 | 1.65 (1.23, 2.07)* | 1.82 (1.40, 2.23)* | 1.03 (1.02, 1.03)* | 1.03 (1.02, 1.03)* |
| 70 | 4 | 79.6 | 80.9 | 1.31 (0.87, 1.75)* | 1.41 (0.97, 1.86)* | 1.02 (1.01, 1.02)* | 1.02 (1.01, 1.02)* |
| 70 | 5+ | 92.1 | 92.5 | 0.43 (0.14, 0.73)* | 0.47 (0.18, 0.77)* | 1.00 (1.00, 1.01)* | 1.01 (1.00, 1.01)* |
| 80 | 0/1 | 23.6 | 25.7 | 2.09 (1.62, 2.57)* | 2.25 (1.78, 2.72)* | 1.09 (1.07, 1.11)* | 1.10 (1.07, 1.12)* |
| 80 | 2 | 51.0 | 53.0 | 2.01 (1.49, 2.53)* | 2.23 (1.71, 2.75)* | 1.04 (1.03, 1.05)* | 1.04 (1.03, 1.05)* |
| 80 | 3 | 68.6 | 70.6 | 2.03 (1.58, 2.48)* | 2.19 (1.74, 2.64)* | 1.03 (1.02, 1.04)* | 1.03 (1.03, 1.04)* |
| 80 | 4 | 80.5 | 82.4 | 1.83 (1.42, 2.23)* | 1.94 (1.53, 2.35)* | 1.02 (1.02, 1.03)* | 1.02 (1.02, 1.03)* |
| 80 | 5+ | 91.6 | 92.6 | 1.03 (0.80, 1.25)* | 1.07 (0.84, 1.30)* | 1.01 (1.01, 1.01)* | 1.01 (1.01, 1.01)* |
| 90 | 0/1 | 31.1 | 33.4 | 2.31 (1.22, 3.40)* | 2.51 (1.43, 3.60)* | 1.07 (1.04, 1.11)* | 1.08 (1.04, 1.12)* |
| 90 | 2 | 57.1 | 59.0 | 1.90 (0.89, 2.90)* | 2.14 (1.14, 3.14)* | 1.03 (1.02, 1.05)* | 1.04 (1.02, 1.06)* |
| 90 | 3 | 71.9 | 74.2 | 2.33 (1.50, 3.17)* | 2.49 (1.65, 3.32)* | 1.03 (1.02, 1.04)* | 1.03 (1.02, 1.05)* |
| 90 | 4 | 81.5 | 83.8 | 2.29 (1.50, 3.07)* | 2.41 (1.62, 3.19)* | 1.03 (1.02, 1.04)* | 1.03 (1.02, 1.04)* |
| 90 | 5+ | 91.0 | 92.7 | 1.65 (1.18, 2.12)* | 1.69 (1.22, 2.16)* | 1.02 (1.01, 1.02)* | 1.02 (1.01, 1.02)* |
| **Notes:** | |  |  |  |  |  |  |
| P(2003) and P(2016) correspond to predicted probabilities of the outcome (polypharmacy) from unadjusted logistic regression | | | | | | | |
| Level of MMB = level of multimorbidity (number of conditions), RD = risk (prevalence) difference, RR = risk (prevalence) ratio | | | | | | | |
| Adj = adjusted (for rurality and area-based income quintile) | | | | |  |  |  |
| All 95% confidence intervals are calculated using the delta method (* denotes p<0.05) | | | | | |  |  |
