# Supplementary material for: Sex differences in multimorbidity and polypharmacy trends: A repeated cross-sectional study of older adults in Ontario, Canada
Source: PLoS One. 2021 Apr 26;16(4):e0250567. doi: 10.1371/journal.pone.0250567 (PMC8075196; doi:10.1371/journal.pone.0250567)
Supplement: S6 Table — (DOCX) [file pone.0250567.s006.docx]

**S6 Table: Marginal probabilities of polypharmacy (5+ drug subclasses) and unadjusted and adjusted risk differences and risk ratios, by select ages, level of multimorbidity and sex**

| **Women** |  |  |  |  |  |  |  |
| --- | --- | --- | --- | --- | --- | --- | --- |
| **Age** | **Level of MMB** | **P(2003)** | **P(2016)** | **RD, Unadj** | **RD, Adj** | **RR, Unadj** | **RR, Adj** |
| 70 | 0/1 | 23.7 | 20.6 | -3.11 (-3.39, -2.82)* | -2.89 (-3.18, -2.60)* | 0.87 (0.86, 0.88)* | 0.88 (0.87, 0.89)* |
| 70 | 2 | 52.0 | 45.7 | -6.30 (-6.66, -5.94)* | -5.98 (-6.35, -5.62)* | 0.88 (0.87, 0.89)* | 0.88 (0.88, 0.89)* |
| 70 | 3 | 70.1 | 64.6 | -5.43 (-5.80, -5.06)* | -5.18 (-5.55, -4.81)* | 0.92 (0.92, 0.93)* | 0.93 (0.92, 0.93)* |
| 70 | 4 | 82.9 | 78.7 | -4.24 (-4.63, -3.85)* | -4.09 (-4.48, -3.70)* | 0.95 (0.94, 0.95)* | 0.95 (0.95, 0.96)* |
| 70 | 5+ | 93.6 | 91.3 | -2.32 (-2.58, -2.06)* | -2.29 (-2.55, -2.02)* | 0.98 (0.97, 0.98)* | 0.98 (0.97, 0.98)* |
| 80 | 0/1 | 29.2 | 28.2 | -1.02 (-1.42, -0.62)* | -0.77 (-1.17, -0.36)* | 0.96 (0.95, 0.98)* | 0.97 (0.96, 0.99)* |
| 80 | 2 | 56.6 | 54.2 | -2.40 (-2.77, -2.02)* | -2.12 (-2.50, -1.74)* | 0.96 (0.95, 0.96)* | 0.96 (0.96, 0.97)* |
| 80 | 3 | 72.6 | 70.8 | -1.82 (-2.14, -1.50)* | -1.62 (-1.94, -1.30)* | 0.97 (0.97, 0.98)* | 0.98 (0.97, 0.98)* |
| 80 | 4 | 83.6 | 82.1 | -1.47 (-1.76, -1.18)* | -1.32 (-1.62, -1.03)* | 0.98 (0.98, 0.99)* | 0.98 (0.98, 0.99)* |
| 80 | 5+ | 92.9 | 92.6 | -0.30 (-0.47, -0.13)* | -0.25 (-0.42, -0.08)* | 1.00 (0.99, 1.00)* | 1.00 (1.00, 1.00)* |
| 90 | 0/1 | 35.3 | 37.2 | 1.86 (0.98, 2.74)* | 2.12 (1.25, 3.00)* | 1.05 (1.03, 1.08)* | 1.06 (1.03, 1.09)* |
| 90 | 2 | 61.1 | 62.5 | 1.38 (0.68, 2.08)* | 1.62 (0.91, 2.32)* | 1.02 (1.01, 1.03)* | 1.03 (1.01, 1.04)* |
| 90 | 3 | 75.0 | 76.3 | 1.24 (0.68, 1.80)* | 1.42 (0.86, 1.98)* | 1.02 (1.01, 1.02)* | 1.02 (1.01, 1.03)* |
| 90 | 4 | 84.2 | 85.0 | 0.89 (0.38, 1.39)* | 1.03 (0.52, 1.54)* | 1.01 (1.00, 1.02)* | 1.01 (1.01, 1.02)* |
| 90 | 5+ | 92.1 | 93.7 | 1.60 (1.31, 1.89)* | 1.66 (1.37, 1.95)* | 1.02 (1.01, 1.02)* | 1.02 (1.01, 1.02)* |
| **Men** |  |  |  |  |  |  |  |
| **Age** | **Level of MMB** | **P(2003)** | **P(2016)** | **RD, Unadj** | **RD, Adj** | **RR, Unadj** | **RR, Adj** |
| 70 | 0/1 | 15.3 | 17.2 | 1.87 (1.61, 2.13)* | 1.98 (1.72, 2.24)* | 1.12 (1.10, 1.14)* | 1.13 (1.11, 1.15)* |
| 70 | 2 | 41.3 | 43.7 | 2.41 (2.01, 2.80)* | 2.60 (2.20, 2.99)* | 1.06 (1.05, 1.07)* | 1.06 (1.05, 1.07)* |
| 70 | 3 | 61.6 | 63.9 | 2.28 (1.85, 2.70)* | 2.45 (2.03, 2.88)* | 1.04 (1.03, 1.04)* | 1.04 (1.03, 1.05)* |
| 70 | 4 | 77.0 | 78.8 | 1.85 (1.39, 2.31)* | 1.97 (1.51, 2.43)* | 1.02 (1.02, 1.03)* | 1.03 (1.02, 1.03)* |
| 70 | 5+ | 91.0 | 91.8 | 0.77 (0.46, 1.07)* | 0.81 (0.51, 1.12)* | 1.01 (1.01, 1.01)* | 1.01 (1.01, 1.01)* |
| 80 | 0/1 | 21.7 | 23.8 | 2.15 (1.70, 2.61)* | 2.30 (1.85, 2.75)* | 1.10 (1.08, 1.12)* | 1.11 (1.08, 1.13)* |
| 80 | 2 | 48.2 | 50.6 | 2.41 (1.90, 2.91)* | 2.63 (2.12, 3.14)* | 1.05 (1.04, 1.06)* | 1.05 (1.04, 1.07)* |
| 80 | 3 | 66.2 | 68.6 | 2.46 (2.01, 2.90)* | 2.63 (2.18, 3.07)* | 1.04 (1.03, 1.04)* | 1.04 (1.03, 1.05)* |
| 80 | 4 | 78.8 | 81.1 | 2.27 (1.87, 2.68)* | 2.39 (1.99, 2.80)* | 1.03 (1.02, 1.03)* | 1.03 (1.03, 1.04)* |
| 80 | 5+ | 90.9 | 92.3 | 1.41 (1.19, 1.63)* | 1.46 (1.24, 1.68)* | 1.02 (1.01, 1.02)* | 1.02 (1.01, 1.02)* |
| 90 | 0/1 | 29.7 | 32.0 | 2.30 (1.22, 3.37)* | 2.49 (1.42, 3.57)* | 1.08 (1.04, 1.11)* | 1.08 (1.05, 1.12)* |
| 90 | 2 | 55.2 | 57.5 | 2.31 (1.32, 3.30)* | 2.56 (1.58, 3.55)* | 1.04 (1.02, 1.06)* | 1.05 (1.03, 1.07)* |
| 90 | 3 | 70.5 | 73.0 | 2.56 (1.74, 3.38)* | 2.72 (1.90, 3.54)* | 1.04 (1.02, 1.05)* | 1.04 (1.03, 1.05)* |
| 90 | 4 | 80.6 | 83.2 | 2.61 (1.86, 3.36)* | 2.73 (1.98, 3.49)* | 1.03 (1.02, 1.04)* | 1.03 (1.02, 1.04)* |
| 90 | 5+ | 90.8 | 92.8 | 2.03 (1.60, 2.46)* | 2.07 (1.64, 2.51)* | 1.02 (1.02, 1.03)* | 1.02 (1.02, 1.03)* |
| **Notes:** | |  |  |  |  |  |  |
| P(2003) and P(2016) correspond to predicted probabilities of the outcome (polypharmacy, 5+ drug subclasses) from unadjusted logistic regression | | | | | | | |
| Level of MMB = level of multimorbidity (number of conditions), RD = risk (prevalence) difference, RR = risk (prevalence) ratio | | | | | | | |
| Adj = adjusted (for rurality and area-based income quintile) | | | | |  |  |  |
| All 95% confidence intervals are calculated using the delta method (* denotes p<0.05) | | | | | |  |  |
