# Supplementary material for: Sex differences in multimorbidity and polypharmacy trends: A repeated cross-sectional study of older adults in Ontario, Canada
Source: PLoS One. 2021 Apr 26;16(4):e0250567. doi: 10.1371/journal.pone.0250567 (PMC8075196; doi:10.1371/journal.pone.0250567)
Supplement: S7 Table — (DOCX) [file pone.0250567.s007.docx]

**S7 Table:** Most frequently dispensed drug subclasses for women and men (age ≥66 years) in Ontario, Canada in 2003 with polypharmacy and

hyper-polypharmacy, and absolute / percent change between 2003 - 2016.

| **Women** | **Polypharmacy** | | |  | **Hyper-polypharmacy** | | |
| --- | --- | --- | --- | --- | --- | --- | --- |
|  | **2003 Prevalence**  **N=532,374** | **Absolute change**  **2003-2016** | **Percent change 2003-2016** |  | **2003 Prevalence**  **N=230,126** | **Absolute change**  **2003-2016** | **Percent change 2003-2016** |
| CORTICOSTEROIDS, PLAIN^a^ | 41.8% | -2.0 | -4.8% | CORTICOSTEROIDS, PLAIN^a^ | 55.6% | -3.0 | -5.4% |
| DIURETICS | 39.8% | -7.6 | -19.2% | DIURETICS | 50.5% | -8.1 | -16.1% |
| ACE INHIBITORS | 38.0% | -10.0 | -26.3% | NON-STEROIDAL ANTI-INFLAMMATORY: NON-ASA BASE^b^ | 46.6% | -20.4 | -43.9% |
| NON-STEROIDAL ANTI-INFLAMMATORY: NON-ASA BASE^b^ | 37.8% | -16.3 | -43.0% | BENZODIAZEPINE DERIVATIVES^b^ | 46.4% | -17.0 | -36.7% |
| BENZODIAZEPINE DERIVATIVES^b^ | 34.5% | -13.2 | -38.1% | NARCOTICS: OPIATE AGONISTS | 45.6% | -3.8 | -8.3% |
| CALCIUM BLOCKERS | 33.6% | +1.4 | 4.1% | ACE INHIBITORS | 45.5% | -13.4 | -29.5% |
| ANTILIPEMIC: STATINS | 33.5% | +23.0 | 68.5% | CALCIUM BLOCKERS | 42.9% | +0.6 | 1.3% |
| NARCOTICS: OPIATE AGONISTS | 31.8% | -3.7 | -11.7% | ANTILIPEMIC: STATINS | 38.8% | +25.1 | 64.5% |
| BETA-BLOCKERS | 30.2% | +0.5 | 1.8% | BETA-BLOCKERS | 36.8% | +3.3 | 9.0% |
| BISPHOSPHONATES^b^ | 25.3% | -2.9 | -11.4% | PROTON PUMP INHIBITORS | 32.1% | +28.7 | 89.5% |
| **Men** | **Polypharmacy** | | |  | **Hyper-polypharmacy** | | |
|  | **2003 Prevalence**  **N=346,851** | **Absolute change 2003-2016** | **Percent change2003-2016** |  | **2003 Prevalence**  **N=135,149** | **Absolute change**  **2003-2016** | **Percent change 2003-2016** |
| ACE INHIBITORS | 48.2% | -9.3 | -19.2% | CORTICOSTEROIDS, PLAIN^a^ | 57.2% | -5.6 | -9.7% |
| ANTILIPEMIC: STATINS | 43.5% | +26.8 | 61.6% | ACE INHIBITORS | 56.0% | -13.5 | -24.1% |
| CORTICOSTEROIDS, PLAIN^a^ | 42.4% | -4.6 | -10.9% | ANTILIPEMIC: STATINS | 49.0% | +27.2 | 55.6% |
| BETA-BLOCKERS | 35.5% | +1.6 | 4.4% | DIURETICS | 46.6% | -7.5 | -16.1% |
| DIURETICS | 34.1% | -6.7 | -19.5% | NARCOTICS: OPIATE AGONISTS | 44.5% | -5.2 | -11.6% |
| CALCIUM BLOCKERS | 33.0% | +0.2 | 0.6% | BETA-BLOCKERS | 42.5% | +5.2 | 12.3% |
| NON-STEROIDAL ANTI-INFLAMMATORY: NON-ASA BASE^b^ | 32.1% | -13.0 | -40.4% | CALCIUM BLOCKERS | 41.6% | -0.5 | -1.2% |
| NARCOTICS: OPIATE AGONISTS | 31.2% | -4.7 | -15.1% | NON-STEROIDAL ANTI-INFLAMMATORY: NON-ASA BASE^b^ | 39.2% | -16.4 | -41.9% |
| BENZODIAZEPINE DERIVATIVES^b^ | 24.4% | -11.1 | -45.6% | BENZODIAZEPINE DERIVATIVES^b^ | 36.2% | -16.3 | -45.1% |
| NON-STEROIDAL ANTI-INFLAMMATORY: ASA BASE^b^ | 23.6% | -20.9 | -88.8% | CORONARY VASODILATORS^b^ | 32.2% | -16.4 | -50.7% |

**Notes:** ^a^ Includes systemic products (e.g., oral) and products for local use. ^b^ Not included on top 10 subclasses in 2016
