# Supplementary material for: Sex differences in multimorbidity and polypharmacy trends: A repeated cross-sectional study of older adults in Ontario, Canada
Source: PLoS One. 2021 Apr 26;16(4):e0250567. doi: 10.1371/journal.pone.0250567 (PMC8075196; doi:10.1371/journal.pone.0250567)
Supplement: Attachment — Submitted filename: MM-Polypharm_PLOS ONE_Response to Reviewers_March 19 2021.docx [file pone.0250567.s009.docx]

**Response Letter**

**Manuscript # PONE-D-21-02920**

**Manuscript Title:** Sex differences in multimorbidity and polypharmacy trends: a repeated cross-sectional study of older adults in Ontario, Canada

**Reviewer #1:**

**Major Issues**

*1. The documents we received do not contain any information describing the study population or the distributions of the analyzed variables. The Table 1 mentioned in the text is unfortunately not included in the supplementary material. The absence of this information also means that points 13,14 and 15 of the STROBE statement are not fulfilled. Could the authors please add this information?*

**Response:** Our Table 1 provides descriptive data on the characteristics of our study populations of older women and men examined in 2003 and 2016. It was included in the manuscript file that we originally submitted to the journal (on pg. 26). With our resubmission, we have moved all our Tables to follow their first mention in the body of the text. This will hopefully make them easier to locate and review. We have also provided a new Supplemental Table (see S3 Table) that summarizes the descriptive characteristics for the total non-stratified (by sex) population of older adults at both time periods.

*2. The authors have compared two different cohorts (all older than 65 years in 2003 were compared with all older than 65 years in 2016)? From our point of view, this warrants more detailed discussion how bias could have been introduced by comparing different cohorts from different points of time.*

**Response:** We are not clear as to the types of bias being referred to by this comment. We agree that the two cohorts of older adults (2003 vs 2016) will have been exposed to varying cohort and period effects that may affect estimates of multimorbidity and polypharmacy (assessed among those aged 66+ years at the two time periods). However, many of these cohort and/or period effects may help to explain some of the changes observed in multimorbidity and polypharmacy levels over time (i.e., they represent potentially explanatory factors for relative/absolute increases or decreases in chronic illness and medication use, e.g., increasing prevalence of obesity in younger and middle-aged adults over time). Such explanatory factors are not sources of bias affecting our study findings.

However, there are other factors that may represent potential sources of bias when comparing multimorbidity and polypharmacy estimates derived from administrative health databases for two different time periods (e.g., changes in the detection, diagnosis and coding of chronic conditions; e.g., shifts in the prescription vs nonprescription status of medications and variation over time in the coverage of medications by the provincial public drug insurance program). We had commented briefly on these potential sources of bias in our original submission, but we have expanded this section to further address this reviewers’ comment (see pg. 23, lines 353-358).

*3. Could the authors please elaborate on their choice of chronic diseases as other chronic diseases, with many drugs used in therapy, such as HIV/AIDS or peripheral vascular occlusive disease, were not considered?*

**Response:** Thank you for this comment. In our original submission, we included background information to support our choice of chronic diseases (for our multimorbidity measure) in our Supplemental S2 Table. To address this comment, we have now incorporated some of this information in the text of our Methods section (see pg. 7, lines 120-124).

*4. Why were some relevant ICD codes (e.g., for atrial fibrillation I48.2 or for osteoporosis M80) not included, although from our point of view they would be important for the correct identification of the diseases considered. Generally, could the authors comment on how the (validated) code sets were derived?*

**Response:** Over the years as we have worked on our multimorbidity projects with these same databases, we have attempted to use codes and case ascertainment algorithms that were either (i) validated by research teams working within the ICES organization (known as existing ICES validated chronic disease cohorts) or (ii) derived from original ICES Reports and Atlases (see our S2 Table). The original codes and algorithms developed were also designed based on what was known at the time and to ensure alignment of ICD-9 and comparable ICD-10 codes. This allowed for some consistency and comparability across related papers reporting on the prevalence of selected chronic diseases and multimorbidity for Ontario.

For the chronic disease cohorts at ICES, there have been some minor alterations in the codes (e.g., refinement to codes as new research and validation efforts arise) and we have adopted these changes for these cohorts (and used them to derive our multimorbidity estimates for 2003 and 2016). Regarding the specific conditions mentioned by this reviewer (atrial fibrillation and osteoporosis), the codes and algorithms we used were consistent with those originally developed for our earlier multimorbidity work. We acknowledge that other research studies may suggest alternative codes or algorithms, so it is possible that our data may represent an underestimate of these conditions – though there is also considerable variability in the diagnostic approaches employed in the literature. We have commented on the potential for misclassification bias in our Limitation section (pg. 23, lines 353-356). Further, previous research has shown that estimates of multimorbidity often remain robust to variation in the number of conditions as long as a minimum number of prevalent (common) conditions are considered (e.g., 12 or more) (see for example: van den Akker M, et al. Multimorbidity in general practice: prevalence, incidence, and determinants of co-occurring chronic and recurrent diseases. J Clin Epidemiol. 1998;51(5):367–75.; Fortin M, et al. A systematic review of prevalence studies on multimorbidity: toward a more uniform methodology. Ann Fam Med. 2012;10(2):142–51; Diederichs C, et al. The measurement of multiple chronic diseases–a systematic review on existing multimorbidity indices. J Gerontol A Biol Sci Med Sci. 2011;66(3):301–11.).

*5. How did the authors deal with the fact that in the long period from 2003 to 2016, new drugs were steadily approved for the treatment of some of the classes they considered (in atrial fibrillation, DOAKs; in diabetes, dulaglutide, etc.)? If these newly approved drugs were not taken into account, this could distort the results, since new drug classes and combination options for the treatment of one and the same disease (and thus a large number of drugs) only exist in the late cohort.*

**Response:** We agree that there will have been new drugs available for the treatment of selected conditions over time. However, we do not see how this would distort the results of our study. Rather, the availability of new treatments and a shift to the use of combination treatments for a particular condition between 2003 and 2016 could represent plausible explanations for any observed increase in polypharmacy estimates. In our Discussion section (pg. 22, lines 330-337), we provided several potential explanations for the increase in polypharmacy observed among older men over time, including more timely diagnosis and treatment of chronic conditions and greater availability, access and/or acceptance of effective treatments.

To further explore polypharmacy trends based on drug subclass rather than unique drug names, we have incorporated further sensitivity analyses where we modeled polypharmacy defined as ≥5 unique drug subclasses (see new S6 Table). The drug database available at ICES uses a modified American Hospital Formulary Service (AHFS) classification scheme to identify drug subclass. The findings from these analyses did not vary in any meaningful way from our primary models for polypharmacy based on ≥5 unique drug names (shown in Table 2).

How the observed changes in polypharmacy and hyper-polypharmacy relate to the appropriateness of treatment decisions (both in 2003 and 2016) is a more complex and different research question that requires further research. We have expanded our exploration of this issue in our Discussion (pg. 24, lines 374-384).

*6. Which drugs were considered in the study to determine polypharmacy? If all medications were considered on an ATC basis, this could distort the results, because also sporadically prescribed treatments such as antibiotics would have been used to determine polypharmacy.*

**Response:** As noted in our Methods section, we included all outpatient drug prescriptions (i.e., excluding inpatient hospital stay prescriptions) filled by Ontario residents aged 66+ years, and reimbursed by the provincial public drug insurance program in the 1 year prior to the index dates. The only outpatient prescription claims excluded were those where the subclass was not identified, not applicable (e.g., contact lens solution), a pharmacy service fee or a device (not a drug). Given we included all unique drugs dispensed during the 1 year prior to each of the index dates and consistent methodological approaches for both time periods, we do not believe that our findings would have been meaningfully distorted by medications (e.g., antibiotics) used on a sporadic basis.

**Reviewer #2:**

*Overall well conducted and reported cross sectional analysis of a population based dataset looking at age and sex specific trends in both multimorbidity and polypharmacy.*

**Response:** Thank you for these positive comments on the strengths of our study.

*1. It is difficult to infer the potential meaning of the result, as no explicit potentially inappropriate medication measures were applied to assess the quality of prescribing at both time points. The authors conclude that the development of specific male targeted deprescribing interventions may be an approach to address the rise in the prevalence of polypharmacy in men over the 13 year period. However, is it possible that this rising prevalence may represent improved prescribing and reduced potentially inappropriate omissions?*

**Response:** The aim of our study was not to examine the quality of prescribing among older adults in 2003 and 2016. As we noted in our response to Reviewer #1 (item #5), identifying the appropriateness of observed changes in polypharmacy prevalence (by age, sex and multimorbidity level) requires further research. However, as noted in our Introduction and Discussion sections, there is significant value in understanding trends in polypharmacy and hyper-polypharmacy (across age, sex and multimorbidity levels) given the strong associations between increasing drug number and the complexity of the drug regimen (and thus treatment burden faced by an older adult that may lead to non-adherence and poor health outcomes) and risk for adverse drug-drug and drug-disease interactions and adverse drug-related events. We agree with this Reviewer that the text of our Conclusion section was too strongly worded (given that the increase in polypharmacy noted for men may in fact reflect appropriate and improved chronic disease management). As noted in our response to Reviewer #1 (item #5) we have revised the text of this section of our Discussion (pg. 24, lines 374-384).

*2. Methods: Line 137. Should the year be 2016 Vs 2003, (Not 2016 Vs 2013)?*

**Response:** Thank you for noting this, we have corrected this in our manuscript.

*3. Results: Please include abbreviations at the end of tables.*

**Response:** Thank you for this comment. In response, we have spelled out in full most of the previous abbreviations (e.g., in the body of the Table) and provided relevant definitions at the end of the Tables for the few abbreviations that remained.

*4. Results: I feel more detail is needed on the age profile of the population cohort at both time points rather than just the median age with interquartile range, for example include the proportion and number of people in various age categories. Could some of the difference in 2016 be explained by changes in male life expectancy? A Danish prospective cohort reported (albeit over a shorter time period) reported an increase in the per person number of medicines in both sexes and that those who exited the study prematurely due to death had a higher number of medicines at entry and a greater increase in the number of medicines up to the time period before death (1).*

**Response:** As suggested, we have included the distribution for age group in our Table 1 (and in S3 Table). Understanding sex differences in life expectancy and in healthy life expectancy over time represents an interesting area of research. Unfortunately, we are not able to comment on the relevance of any such differences as we conducted a repeated cross-sectional study of older adults. We have included some additional text to our Discussion commenting on these as areas for further research (pg. 21, lines 305-310)

*5. Results: Would it be possible to include a similar figure to figure 1 showing the proportion with 0, 1, 2, 3 etc. medicines at the various age cuts. I think visualising the actual proportion for each number of medicines at both time points would help give the reader a sense of the magnitude of the change over the 13-year period?*

**Response:** We believe this comment relates to our Figure 2 (not Figure 1). We prefer to keep our Figure 2 as it is given it clearly shows the proportion of older women and men with our main outcome measures (i.e., polypharmacy and hyper-polypharmacy) by age and time period. We have added the proportion with 0, 1, 2, 3, 4 and 5+ dispensed medications to our Table 1.

*6. Results: Line 219, corticosteroids use seems very high. Can you specify if these were systemic or topical/inhaled?*

**Response:** The corticosteroid (plain) subclass was largely made up of products for local use (e.g., topical). Systemic products taken primarily by mouth were also included in this subclass but made up less than 20% of overall use. We have added a comment about this as a footnote to our Table 4.

*7. Discussion: The authors have clearly stated it is not possible to assess causation due to the cross sectional design but I feel possible reasons for these sex differences should be explored a little more particularly with respect to potentially inappropriate prescribing as this would add weight to the finding and might guide more specific recommendations for further research.*

**Response:** Please see our response to item # 1 above. To address this further, we have provided further comment about the observed sex-differences to our Discussion (pg. 21, lines 305-310).

*8. Discussion: I agree it would be interesting to correlate the findings with changes in PIP, which has generally declined when increases in polypharmacy are accounted for (2). Perhaps more men had potentially inappropriate omissions and more women had potentially inappropriate prescriptions (especially related to hypnotic and analgesia use). Perhaps these changes reflect improvements in prescribing over the 13-year study period.*

**Response:** Please see our response to item #1 & #7 above.

**Reviewer #3:**

Please note, there were some statements by this Reviewer that appeared to be incomplete or were otherwise difficult to follow – we have done our best to address the text that was provided.

*1. In Backgrounds section, the main ideas are related with (i) an upward trend in multimorbidity, (ii) a correlation between coexistence of chronic diseases and medication balance (polypharmacy) and (iii) the lack of differences between sex groups in previous publications. However, too many references have been used and it is quite difficult to follow the flow of these ideas. Lines 66-68 can be answered with any of the systematic reviews driven by Prados-Torres (DOI:10.1016/j.jclinepi.2013.09.021) or C. Violan (*[*https://doi.org/10.1371/journal.pone.0102149*](https://doi.org/10.1371/journal.pone.0102149)*). In my opinion, last sentence of this section (line84-85) should be placed in Discussion section being developed more accurately.*

**Response:** Thank you but we do not believe that we have used too many references to adequately support the statements made in our Introduction section. We prefer to reference original source studies (specific to our target population of interest) to support these statements rather than referencing systematic reviews that include different study populations and settings.

We agree with this Reviewer that the last part of the last sentence of our Introduction should be removed and have done so.

*2. In Methods section: it should be defined the acronyms ICES (line 97)I could not find it in main document or supplementary files. Google helped me to know that is an Institution with health information from Ontario. I recommend to describe a brief description or add a reference because this info is added in “data availability” at the end of the document. Related with inclusion criteria, I was wondering if 65 years are included or people are older (66 years), consider using > or ≥, or +65 years as it was used to describe multimorbidity and medication use (homogenized).*

**Response:** As suggested, we have provided further details regarding the ICES organization (see pg. 6, lines 103-107).

With regard to the lower age range for our study populations, this was restricted to persons aged 66 years and older as we required a 1-year look back period from index date to derive our measures of polypharmacy given the prescription drug claims data (ODB) are only available for persons aged 65 years and older. We have clarified this in our Methods section (see pg. 6, lines 95-96) and we have replaced all occurrences of “>65” with “66+ years” in our manuscript and Tables.

*3. Results sections - I suggest some points.
oIt can be considered a row in Table 1 with relative differences between 2016 and 2003 to evaluate trends between both time points.*

*oI suggest in Line 192 and 198 homogenize “those with no or 1 condition” by “0/1 condition”*

*o Results of polypharmacy and hyperpolypharmacy from 2003 (s5) is quite confusing. It is said that NSAIDs and BDZ are declined, I guess they are “declined compared to hyperpolypharmacy group”. May be confusing the meaning of this findings. What about other differences like corticosteroids or diuretics? They seemed to have the same trend and they are not mention.*

**Response:** (i) For the first point, we are not clear on what the Reviewer is asking for us to include in Table 1 – is the request for an additional row? (column?) with estimates of relative differences between 2003 and 2016? And if so, for which measures? Adding 2 additional columns (one for women and one for men) to illustrate relative differences in all of these measures we feel would overly crowd and complicate Table 1. Because of this, we have elected to keep our Table 1 as is.

(ii) For the second point, we prefer to keep the text (“…those with no or 1 condition…”) as written for purposes of clarity.

(iii) For the third point, we agree this section and the Tables were not as clear as they could be and we have revised both the text of our Results section (see pg. 17, lines 240-252) and our Table 4 (2016) and S7 Table (2003). In both our Table 4 and S7 Table we have also included additional columns to clearly illustrate the absolute and percent change in the most common subclasses for women and men with polypharmacy and hyper-polypharmacy (comparing changes from 2003 to 2016).

*4. In Discussion section, the text brings all the information into a coherent whole, connecting the findings with the aim of the study. However, authors interpretation is missing at some point. For example, why NSAIDs and BDZ are common in older people with hyperpolypharmacy rather than polypharmacy, they are included in STOP/START criteria and they are still very common. Besides, scarcely clinical meaning is discussed. Women have a longer expectancy of living, they have different multimorbidity patters compared with men because of gender-related diseases and lifestyle (for instance, smoking or alcohol habit (DOI: 10.1016/j.jclinepi.2013.09.021). In my opinion, discussion results are quite in the surface and could be improved.*

**Response:** To provide further reflection on some of the key messages relevant to our study, we have added new text to our Introduction (pg. 4, lines 67-71) and our Discussion (pg. 21, lines 305-310) to highlight the rationale for examining sex-differences in multimorbidity and polypharmacy trends as well as some interesting hypotheses to pursue in future research.

Regarding the comment about the START/STOPP criteria – we are aware these agents are included in these and other criteria (e.g., Beers) for defining potentially inappropriate drug use in older adults but as noted in our comment to Reviewer 2 (item #1), assessing the appropriateness of polypharmacy/hyper-polypharmacy was not an objective of our study. Our study was a repeated cross-sectional study, not a prospective cohort, this limits our ability to draw conclusions about causal relationships and we have been cautious in the interpretation of our study findings for this reason.

*5. On the other hand, it has been interesting to work with most common chronic conditions because it is easier to understand the relation of data. However, heterogeneity in chronic condition combination is not represented in this article. In limitation section it should be considered the few number of chronic conditions considered. Multimorbidity is probably higher because of this limitation (DOI: 10.1016/j.jclinepi.2013.09.021).*

**Response:** Please see our comment to Reviewer #1 (items # 3 & 4). In our original submission we noted the need for research on sex-specific trends in chronic condition clusters in the limitation section of our Discussion (pg. 23, lines 361-363). An exploration of sex-differences in chronic disease or multimorbidity clusters was not the aim of our study. Also note that we have included a statement about our multimorbidity estimates (i.e., that they may vary from studies using different or more conditions) in our limitation section (pg. 23, lines 353-356).

*6. Finally, the conclusion should be more specific. It might end with a strong statement but is quite redundant with results found. It contains references and in my opinion, references should be placed in discussion section. This section need to emphasize contribution of your work and may be it implications for future research.*

**Response:** We have revised the text of our conclusions to clarify potential interpretations of our findings and future research priorities (pg. 24, lines 374-384). As noted in our response to item #4 above, we believe it is important that our statements also accurately reflect our study design and the data examined.
